# Presence of cholesterol oxides in milk chocolates and their correlation with milk powder freshness

**DOI:** 10.1371/journal.pone.0264288

**Published:** 2022-03-21

**Authors:** Davide Risso, Valerio Leoni, Federico Canzoneri, Matteo Arveda, Rosanna Zivoli, Andrea Peraino, Giuseppe Poli, Roberto Menta

**Affiliations:** 1 Soremartec Italia Srl, Ferrero Group, Alba, Italy; 2 Laboratory of Clinical Chemistry, Hospital of Desio and Monza, ASST-Monza, School of Medicine and Surgery, University of Milano Bicocca, Milan, Italy; 3 Department of Clinical and Biological Sciences, University of Torino, San Luigi Hospital, Turin, Italy; Louisiana State University Health Sciences Center, UNITED STATES

## Abstract

Cholesterol oxidation products (COPs) of non-enzymatic origin are mainly found in meat, fish, eggs and milk, mostly originating from the type of feeding, processing and storage. To verify the significance of COPs as biomarkers of cholesterol autoxidation and milk freshness, we quantified them in chocolates containing whole milk powders (WMPs) of increasing shelf-lives (i.e. 20, 120, and 180 days). Non-enzymatic total COPs (both free and esterified) ranged from 256.57 ± 11.97 to 445.82 ± 11.88 ng/g, increasing proportionally to the shelf-life of the WMPs, thus reflecting the ingredients’ freshness. Based on the expected theoretical COPs, the effect of processing was quantitatively less significant in the generation of oxysterols (41–44%) than the contribution of the autoxidation of the WMPs over time (56–59%), pointing to the shelf-life as the primary determinant of COPs. Lastly, we quantified COPs of major commercial milk chocolates on the Italian market, which followed a similar distribution (from 240.79 ± 11.74 to 475.12 ± 12.58 ng/g). Although further replications of this work are needed, this study reports preliminary results and a practical example of a first application of non-enzymatic COPs as markers to further quantify and characterize the nutritional quality and freshness, not only of ingredients but also of composite products.

## Introduction

Cholesterol, one of the most abundant individual lipid molecular species within the human body, is an essential molecule for mammalian cells, playing several roles that ensure normal cells physiology. Besides guaranteeing membrane structure and fluidity, cholesterol is also a precursor of several hormones -both sex (e.g. testosterone and estrogens) and steroid (e.g. cortisol and aldosterone)- vitamin D, and one of the major lipid constituents of bile [1]. It can be obtained from two sources, namely synthesized *de novo* by hepatocytes or from the diet [2]. Because of the presence of Δ5 unsaturated bonds, cholesterol is particularly subject to oxidation when exposed to processing and/or storage procedures that can compromise the nutritional properties of foodstuffs including heating, spray-drying and prolonged exposure to oxygen [3]. This leads to the generation of non-enzymatic oxysterols, or cholesterol oxidation products (COPs), some of which, in particular 7β-hydroxycholesterol (7βOHC) and 7-ketocholesterol (7KC), have received much attention because at relatively high concentrations might induce a cytotoxic effect and play a role in the pathology of a variety of diseases [4, 5]. Considering that non-negligible amounts of cholesterol are also found in feed, COPs of foodstuff of animal origin may also be affected by the sterol profile of the animal feed [6]. In addition, it has been shown how chemical reactions between COPs and reactive amino acids can occur [7]. Lastly, sterol esterification, a cellular process that limits the amount of free (unesterified) cholesterol in cell membranes, is another important factor that plays a role in governing the susceptibility towards oxidation, as esterified sterols may possess a higher oxidative stability, compared to free ones [8, 9]. Although no estimation of dietary exposure and intake assessment is present on COPs yet [4], a reduced consumption has been suggested, either through the reduction of their production during the food chain or by application of a shorter shelf-life [4, 10]. For comparative purposes, it is worth mentioning that, for phytosterol oxidation products (POPs), it was estimated a broad dietary exposure up to 130 mg/d, depending on the assumed oxidation rate and the enrichment status of the food products [11, 12]. Since no toxicity limit for such compounds has been specified yet, the threshold of toxicological concern (TTC) [13] for each unclassified compound (0.15 μg per person per day) is currently utilized as reference upper limit of intake for such compounds [10].

7βOHC and 7KC have been extensively quantified in several animal-based foods including eggs, meat, fish, cheese, and milk [3, 14]. However, besides infant formulas and a few other exceptions (i.e. biscuits, sweet snacks, and pasta) quantitative data in composite products are scant [15–18]. Other non-enzymatic oxysterols, such 5,6α-epoxycholestanol (α-epoxy), 5,6β-epoxycholestanol (β-epoxy), choles-tan-3β,5α,6β-triol (triol), and oxysterols of both enzymatic and non-enzymatic origin, such 7α-hydroxycholesterol (7αOHC), and 25-hydroxycholesterol (25OHC), have also been identified in animal-based products [3, 14, 19]. The oxysterol of sole enzymatic origin 27-hydroxycholesterol (27OHC), so far has only been reported in concentrations of antiviral potential in bovine colostrum and in lower but still nutritionally relevant amounts in mature milk and milk powders, without being affected by processing of storage [20].

Fresher products, milk and eggs in particular, contain lower COPs than ones stored for longer periods or more intensively processed [19, 21]. For these reasons, such compounds have been presented as promising markers to be monitored to preserve the nutritional value of milk and milk products [20, 22]. Similarly, 7KC especially, has recently been identified as a biomarker of milk-based infant formula manufacturing and potential biomarker for the early detection of inherited disorders related to cholesterol metabolism and of food safety [4, 15, 23].

On these premises, considering the lack of quantitative data on COPs in composite products, this study evaluated for the first time the occurrence of oxysterols in commercial samples of milk chocolate and explored their relationship with ingredients’ freshness through the development of *ad hoc* chocolate tablets formulated with milk powders at different shelf-lives. The main purpose of this preliminary study was to verify the applicability of the total content of total (i.e. free and esterified non-enzymatic) oxysterols as markers of cholesterol autoxidation and milk powder freshness also in composite products, to propose them as objective parameters to substantiate the claims related to milk quality that these products often bear.

## Materials and methods

### Prototype milk chocolate production

The European Union (EU) has established definitions of cocoa and chocolate products and harmonized their labelling in order to enable consumers to make informed choices. Milk chocolates must contain not less than 25% total dry cocoa solids, 14% dry milk solids (obtained by partly or wholly dehydrating whole milk, semi- or full-skimmed milk, cream, or from partly or wholly dehydrated cream, butter or milk fat), 2.5% dry non-fat cocoa solids, 3.5% milk fat, and 25% total fat (cocoa butter and milk fat) [24]. According to this Directive, additional information relating to quality criteria of milk chocolate may also be supplemented, provided that the products contain not less than 30% total dry cocoa solids and 18% dry milk solids obtained by partly or wholly dehydrating whole milk, semi- or full-skimmed milk, cream, or from partly or wholly dehydrated cream, butter or milk fat, including not less than 4,5% milk fat [24].

Milk chocolates were therefore identified as ideal composite product candidates for the purpose of this study, as they are formulated with different types of milk powders and ingredients, in variable but generally important concentrations, with the aim of conferring specific physical and organoleptic properties that are often leveraged to claim and highlight their freshness and/or quality [25–27].

Three prototypes of milk chocolate tablets were prepared according to a master recipe in which only the shelf life of milk powder was considered as a variable. The master recipe was comprised of sugar, whole milk powder (WMP) 23.4%, cocoa butter, cocoa liquor, soy lecithin, and vanillin. Each sample of chocolate was prepared mixing chocolate liquor, sugar, milk and cocoa butter in a mixer at 50°C (Hobart FEG, Ohio, US) for a short time. The mixed ingredients were refined in a 3-roll refiner (Bühler Group, Uzwil, Switzerland) and then conched for 3 hours, at 50°C for the first 90 min and then at 45°C until 180 min. Vanillin and soy lecithin were added in the final conching step. All chocolate samples were manually tempered on a cold granite countertop and molded in plastic moulds. After cooling the moulds were demoulded, packed in a plastic film and stored at 18°C until tested (within 48 hours after collection). To minimize external variables that could influence the results, the three chocolates were produced the same day by the same operator following the same protocol. Chocolate A contained fresh WMP with a shelf-life of 20 days. Chocolates B and C contained the same percentage of WMP from the same supplier, at shelf-life of 120 and 180 days, respectively. WMPs at different shelf-life stages were from different batches but produced under the same industrial conditions and stored at controlled temperatures (20 ± 2°C) and humidity (< 65% relative humidity -RH-) in the dark under non-vacuum conditions. The shortest shelf-life (i.e. 20 days) was chosen on the assumption of a freshest application and inclusion in industrial products, considering the logistics and availability from suppliers. The intermediate shelf-life (i.e. 120 days) was chosen based on previous evidence showing how flavor changes in WMPs tend to occur after 3 months under non-vacuum storage [28]. In absence of regulatory requirements governing the shelf-life of WMPs in Europe, we selected a precautionary 180 days as the longest shelf-life, based on information recommending a maximum shelf-life of 6 months at room temperature [29]. It is important to stress, however, that some industry standards present in other countries such in the case of the US, or literature data, report even higher shelf-lives of 6–9 months or > 1 year, respectively [30, 31]. All the other ingredients were included at the same concentrations in all formulations and were at the same shelf-life. The simulated effect of the processing of the chocolates was based on the theoretical amounts of COPs expected for the three chocolates, starting from the analytical COPs content quantified in the ingredients before their inclusion in the products, knowing their dosage in the recipes.

### Commercial chocolate samples

Commercial samples of 9 milk chocolate tablets at the same shelf-life, identified as industrial representative and best-selling products of such category with comparable ingredient lists, were purchased in Italian supermarkets the same day within March 2021. Samples were then stored in the dark at 4°C until tested (within 48 hours after collection).

### Method validation and determination of oxysterols and cholesterol

To our knowledge, this is the first time that milk chocolates have been analyzed for oxysterols content. Therefore, we adapted previously published methods of oxysterols quantification to this newly analyzed matrix and included validation steps to ensure the reliability of our determinations. More specifically, replicated analysis (n = 10) of milk chocolate resulted in an inter-assay coefficient of variation (CV) as 3.45% for 7βOHC, 2.53% for 7KC, 4.52% for α-epoxy, 3.59% for β-epoxy, 4.86% for triol, 2.57% for 25OHC and 3.81% for 27OHC. Limit of quantification was 0.05 ng for each determined compound. Mean recovery (measured/expected %) on milk chocolate samples (n = 3) spiked with 50 ng of each oxysterols was 101% for 7βOHC, 99.4% for 7KC, 98.2% forα-epoxy, 98.2% for β-epoxy, 99.5% for triol, 96.3% for 25OHC, and 98.9% for 27OHC, respectively.

As described elsewhere [20, 32], oxysterols were determined as follows: to a screw-capped vial sealed with a teflon septum, 250 mg of milk powder dissolved in distilled water or 500 mg of homogenized chocolate were added together with 50 μg of epicoprostanol (Sigma) and 50 ng of deuterium labeled oxysterols (Table 1) as internal standards, and 50 μL of butylated hydroxytoluene (BHT, 5 g/L) and 50 μL of K3-EDTA (10 g/L) to prevent auto-oxidation.

**Table 1:** List of sterols and oxysterols quantified by isotope dilution mass spectrometry.

Full name	Short name	SKU	m/z	RT min
Epicoprostanol*	epiC	1236663	370	11.18
cholesterol	chol	C8667	368	12.02
7β-hydroxycholesterol-d7*	d7-7βOHC	700044P	463	12.59
7β-hydroxycholesterol	7βOHC	700035P	456	12.68
5α,6α-epoxycholestanol-d7*	d7-α-epoxy	700047P	481	13.03
5α,6α-epoxycholestanol	α-epoxy	700032P	474	13.20
5ß,6ß-epoxycholestanol-d7*	d7-β-epoxy	700014P	481	13.31
5ß,6ß-epoxycholestanol	β-epoxy	700033P	474	13.46
5a,6ß-dihydroxycholestanol-d7*	d7-triol	700055P	409	14.46
Cholestane-3ß,5α,6ß-triol	triol	700054P	403	14.52
25-hydroxycholesterol-d6*	d6-25OHC	700053P	137	15.35
25-hydroxycholesterol	25OHC	700019P	131	15.43
7-ketocholesterol-d7*	d7-7KC	700046P	479	15.55
7-ketocholesterol	7KC	700015P	472	15.69
27-hydroxycholesterol-d*	d6-27OHC	700059P	462	16.41
27-hydroxycholesterol	27OHC	700021P	456	16.50

* Internal standards. Cholesterol and Epicoprostanol were purchased by Sigma-Merck. Oxysterols were purchased by Avanti Polar Inc. USA.

Each vial was flushed with argon for 10 min to remove air. Alkaline hydrolysis was allowed to proceed at 4°C overnight with magnetic stirring in the presence of ethanolic 2 M potassium hydroxide solution. After hydrolysis, the sterols were extracted with cyclohexane: 5 ml of cyclohexane were added to each vial, vortexed for 30s and allow to separate at 4°C for 15 minutes. Finally, the vials were centrifuged at 3500 x g for 10 min at 4°C. The upper cyclohexane phase was collected in a clean tube. This step was repeated twice. Thus, 3 mL of the cyclohexane extract were used for cholesterol analysis. The oxysterols were separated from cholesterol and sterols by elution of the remaining 7 mL on SPE cartridge (SI 100 mg columns, Isolute). The cyclohexane extract was dried under argon stream at room temperature. The residue was dissolved in 1 mL of toluene. Silicate cartridges were equilibrated with 3 × 1.0 mL n-hexane. The toluene dissolved samples were loaded onto a silica cartridge and allowed to pass through the column by gravity force at room temperature. The columns were washed with 8 × 1.0 mL 0.5% 2-propanol in n-hexane (v/v) followed by elution of the oxysterols with 5 × 1.0 mL 30.0% 2-propanol in n-hexane (v/v) as previously described [33, 34]. The organic solvents were evaporated under a gentle stream of argon and converted into trimethylsilyl ethers with 100 μL of BSTFA (70°C for 60 min).

Analysis was performed by isotope dilution gas chromatography mass spectrometry (GC-MS) with aB-XLB column (30 m x 0.25 mm i.d. x 0.25 μm film thickness, J&W Scientific Alltech, Folsom, CA, USA) in a HP 6890 NNetwork GC system (Agilent Technologies, USA) connected with a direct capillary inlet system to a quadrupole mass selective detector HP5975B inert MSD (Agilent Technologies, USA). GC system was equipped with a HP 7687 series autosamplers and HP 7683 series injectors (Agilent Technologies, USA).

The oven temperature program was as follows: initial temperature of 180°C was held for 1 min, followed by a linear ramp of 20°C/min to 270°C, and then a linear ramp of 5°C/min to 290°C, which was held for 11 min. Helium was used as carrier gas at a flow rate of 1 mL/min and 1 μL of sample was injected in splitless mode. Injection was carried out at 250°C with a flow rate of 20 mL/min. Transfer line temperature was 290°C. Filament temperature was set at 150°C and quadrupole temperature at 220°C according with the manufacturer indication. Mass spectrometric data were acquired in selected-ion monitoring mode at m/z = 463 for d7-7βOHC, m/z = 456 for 7βOHC (Sigma-Aldrich Inc. USA, SKU: H6891), m/z = 474 for α-epoxy (AnvatiPolar Lipids, Inc. USA, SKU:7000329) and for β-epoxy (AnvatiPolar Lipids, Inc. USA, SKU 700033), m/z = 481 for d7-α-epoxy and d7-β-epoxy, m/z = 403 for triol (AvantiPolar Lipids Inc, SKU: 700054P), m/z = 410 for d7-triol, m/z = 479 for d7-7KC, m/z = 472 for 7KC (Sigma-Aldrich Inc. USA, SKU: C2394), m/z = 131 for 25OHC (Anvatipolar Lipids Inc., SKU: 700019), m/z = 137 for d6-25OHC, m/z = 462 for d6-27OHC and m/z = 456 for 27OHC (Avanti Polar Lipids Inc. USA, SKU: 700021P). Peak integration was performed manually, and oxysterols were quantified from selected-ion monitoring analysis against internal standards using standard curves for the listed sterols [20, 35–37].

Cholesterol was measured by GC-MS as previously published [19, 32], using the same GC program as described above. The 3 mL of the cyclohexane extract for cholesterol analysis were evaporated under a gentle stream of argon and converted into trimethylsilyl ethers with 100 μL of BSTFA (70°C for 60 min). Mass spectrometric data were acquired in selected-ion monitoring mode at m/z = 370 for epicoprostanol (internal standard) and m/z = 368 for cholesterol. Inter-assay CV (n = 10) was 1.34%. Limit of quantification was 0.25 μg. Mean recovery (measured/expected %) on milk chocolate samples (n = 3) spiked with 100 μg was 1.52%.

### Statistical analysis

Continuous variables were inspected and tested to determine whether distributions were normal by Kolmogorov–Smirnov normality test, expressed as mean ± SD and compared using ANOVA with the Scheffé post-test for parametric data, with Holm-Sidak method for All Pairwise Multiple Comparison. Comparison between two groups was performed with two tailed Student’s t-test, and values for statistical significance were set at p < 0.05. All analyses were performed with Sigmastat 3.5 (Sigma-Aldrich, St Louis, MO, USA).

## Results and discussion

### COPs are present in both dairy and plant-based ingredients

To investigate the source of COPs present in the finished product, apart from sugar and vanillin, we analyzed all the ingredients before using them to produce the milk chocolate tablets. In WMPs, out of the seven oxysterols analyzed, the most abundant were 7βOHC and 7KC (86–88% of total non-enzymatic COPs, Table 2), in line with previous findings [20, 38].

**Table 2:** Cholesterol (μg/g) and COPs (ng/g) in the three prototype milk chocolate bars and the ingredients used for their production.

Samples	Cocoa paste	Cocoa butter	Soy lecithin	WMP^1^ 20 days	WMP^1^ 120 days	WMP^1^ 180 days	Milk chocolate A^2^	Milk chocolate B^2^	Milk chocolate C^2^
**Cholesterol**	24.46 ± 0.76	42.69 ± 0.84	30.23 ± 0.76	750.25 ± 6.88^a^	826.38 ± 9.71^b^	865.13 ± 8.39^c^	226.74 ± 5.38	246.80 ± 2.46	222.04 ± 2.62
**7β-hydroxycholesterol (7βOHC)**	38.54 ± 1.01	79.78 ± 1.65	48.16 ± 1.34	180.40 ± 4.17^a^	302.82 ± 14.81^b^	477.3 ± 14.81^c^	124.16 ± 4.43^ab^	160.16 ± 4.31^ac^	216.20 ± 7.6^ad^
**7-ketocholesterol (7KC)**	23.94 ± 0.58	44.47 ± 0.61	39.84 ± 1.75	206.18 ± 8.33^a^	247.45 ± 5.42^b^	293.12 ± 3.34^c^	80.61 ± 3.45^ab^	107.79 ± 4.61^ac^	143–23 ± 2.87^ad^
**5,6α- epoxycholestanol (α-epoxy)**	3.94 ± 0.05	6.07 ± 0.33	4.13 ± 0.16	18.30 ± 0.27^a^	21.87 ± 0.71^b^	30.39 ± 1.61^c^	18.95 ± 0.57^ab^	26.99 ± 0.38^ac^	31.08 ± 0.20^ad^
**5,6β- epoxycholestanol (β-epoxy)**	3.61 ± 0.12	5.57 ± 0.23	0.93 ± 0.03	9.48 ± 0.23^a^	10.71 ± 0.31^b^	15.80 ± 0.52^c^	12.39 ± 1.91^ab^	17.98 ± 0.84^ac^	25.24 ± 0.71^ad^
**cholestan-3β,5α,6β-triol (triol)**	0.11 ± 0.01	0.09 ± 0.01	0.20 ± 0.01	6.07 ± 0.48^a^	14.25 ± 0.26^b^	25.38 ± 0.71^c^	1.58 ± 0.04^ab^	2.25 ± 0.07^ac^	6.05 ± 0.07^ad^
**25-hydroxycholesterol (25OHC)**	18.04 ± 1.03	26.52 ± 0.9	10.60 ± 0.5	28.74 ± 1.72^a^	29.71 ± 1.32^b^	40.86 ± 1.87^c^	18.87 ± 1.56	20.64 ± 1.47	24.02 ± 0.44
**27-hydroxycholesterol (27OHC)**	21.58 ± 0.86	45.16 ± 2.16	9.89 ± 0.46	156.3 ± 2.12	165.55 ± 2.3	150.3 ± 4.12	95.13 ± 4.96	96.68 ± 2.32	114.02 ± 3.40
**Total non-enzymatic COPs** ^**1**^	88.17 ± 2.81	162.50 ± 3.71	103.86 ± 3.8	449.17 ± 15.19^a^	626.80 ± 10.17^b^	882.86 ± 22.86^c^	256.57 ± 11.97^ab^	335.81 ± 11.68^ac^	445.82 ± 11.88^ad^
**Non-enzymatic COPs** ^**1**^ **/cholesterol ratio (ng/μg)**	3.60 ± 0.01	3.81 ± 0.02	3.44 ± 0.06	0.60 ± 0.02^a^	0.76 ± 0.01^b^	1.02 ± 0.02^c^	1.13 ± 0.04^ab^	1.36 ± 0.05^ac^	2.01 ± 0.04^ad^

Values are expressed as means ± SD (n = 3).

Different letters in the same line indicate significant differences (P < 0.05).

1 COPs: cholesterol oxidation products; WMP: whole milk powder.

2 Milk chocolates A, B and C were made with WMPs of 20, 120, and 180 days of shelf-life, respectively.

Non-enzymatic COPs in WMPs increased significantly over time in a reverse manner of the ingredient’s freshness, in agreement with previous results highlighting their role as reliable markers of autoxidation across shelf-life [20, 38, 39]. Fresh (20 day) WMP contained a total of 449.17 ± 15.19 ng/g non-enzymatic COPs that increased to 626.8 ± 10.17 (1.4-fold increase) and 882.86 ± 22.86 ng/g (2-fold increase) in older WMPs of 120 and 180 days, respectively (p < 0.001; Table 2). Similarly, when considering the cholesterol amount present in the three WMPs (average of 813.92 ± 51.13 μg/g), the non-enzymatic COPs/cholesterol ratio increased from 0.60 ± 0.02 to 0.76 ± 0.01 and 1.02 ± 0.02 ng/μg, respectively (p < 0.001; Table 2). As predicted, and previously illustrated, 27OHC did not change significantly according to storage time (p < 0.001; Table 2) [20].

Cocoa butter, cocoa paste, and soy lecithin contained cholesterol amounts of 24.46 ± 0.78, 42.69 ± 0.84 and 30.23 ± 0.76 μg/g, respectively. This is confirmed by previous reports showing how cholesterol, differently from the common belief, is also present in plant-based ingredients, although in much lower amounts [40, 41]. Non-enzymatic COPs were also present, reaching concentrations of 88.17 ± 2.81, 162.50 ± 3.71 and 103.86 ± 3.79 ng/g, with 7βOHC and 7KC being once again the most represented (71–85% of total non-enzymatic COPs). 27OHC was present in concentrations of 21.58 ± 0.86, 45.16 ± 2.16 and 9.89 ± 0.46 ng/g, respectively. However, considering the minute amounts of cholesterol present, the non-enzymatic COPs/cholesterol ratio of 3.6, 3.81 and 3.44 ng/μg is significantly higher than the one observed in animal-based products (p < 0.001; Table 2) [14, 20]. To the best of our knowledge, besides in vegetable oils [42], this is the first report of quantitative values of COPs in other non-animal-based products.

### COPs in prototype milk chocolates are correlated with their milk ingredients freshness

The three milk chocolates tablets prototypes contained an average cholesterol level of 231.86 ± 3.49 μg/g (Table 2), an amount compatible with data present in international food databases for milk chocolates [43]. According to what reported for WMPs at different shelf-lives, significant changes in COPs were observed among milk chocolates A (with WMP of 20 days old), B (with WMP of 120 days), and C (with WMP of 180 days). More specifically, compared to milk chocolate A, total non-enzymatic COPs increased in samples B and C by 1.3 and 1.7-fold, respectively, following an increase ratio comparable to the one of the three WMP ingredients at different shelf-lives (p < 0.001). Similarly, the non-enzymatic COPs/cholesterol ratio increased from 1.13 ± 0.04 in chocolate A, to 1.36 ± 0.05 in chocolate B (1.2-fold) and 2.01 ± 0.04 ng/μg in chocolate C (1.8-fold) (p < 0.001; Table 2). This reflected the ratio observed in the three WMPs (p > 0.05). As in the case of the ingredients, the main non-enzymatic COPs in the three chocolates were 7βOHC and 7KC (80–81% of total non-enzymatic COPs, Table 2). This further strengthens their role as reliable markers of cholesterol autoxidation also in composite products. We then simulated the theoretical amounts of COPs of the three chocolates based on the analytical values of the ingredients used for their production. Based on such, we would have expected a total amount of COPs of 150.15 ± 1.17, 191.69 ± 0.88 and 251.56 ± 1.62 ng/g in chocolates A, B, and C, respectively, deriving mostly (~76%) from WMP, rather than from plant-based ingredients (~24%). Compared to the determined values of 256.57 ± 11.97, 335.81 ± 11.68, and 445.82 ± 11.88 ng/g, these amounts where significantly lower (p < 0.001). When comparing the ratio between the expected and the actual values, however, no significant differences were identified (0.59 ± 0.02, 0.57 ± 0.02, and 0.56 ± 0.01; p > 0.05). This suggests that the effect of processing in the generation of COPs is constant over the production of the chocolates and has less influence, compared to the amount of oxysterols deriving from the auto-oxidation of shelf-life of the WMPs. Such findings agree with a previous report on POPs in other composite products (i.e. spreadable creams), where their presence was attributed to the oils used as raw materials, rather than to the technological processes to produce them [44].

### Occurrence of oxysterols in commercial chocolate samples

**Fig 1:**
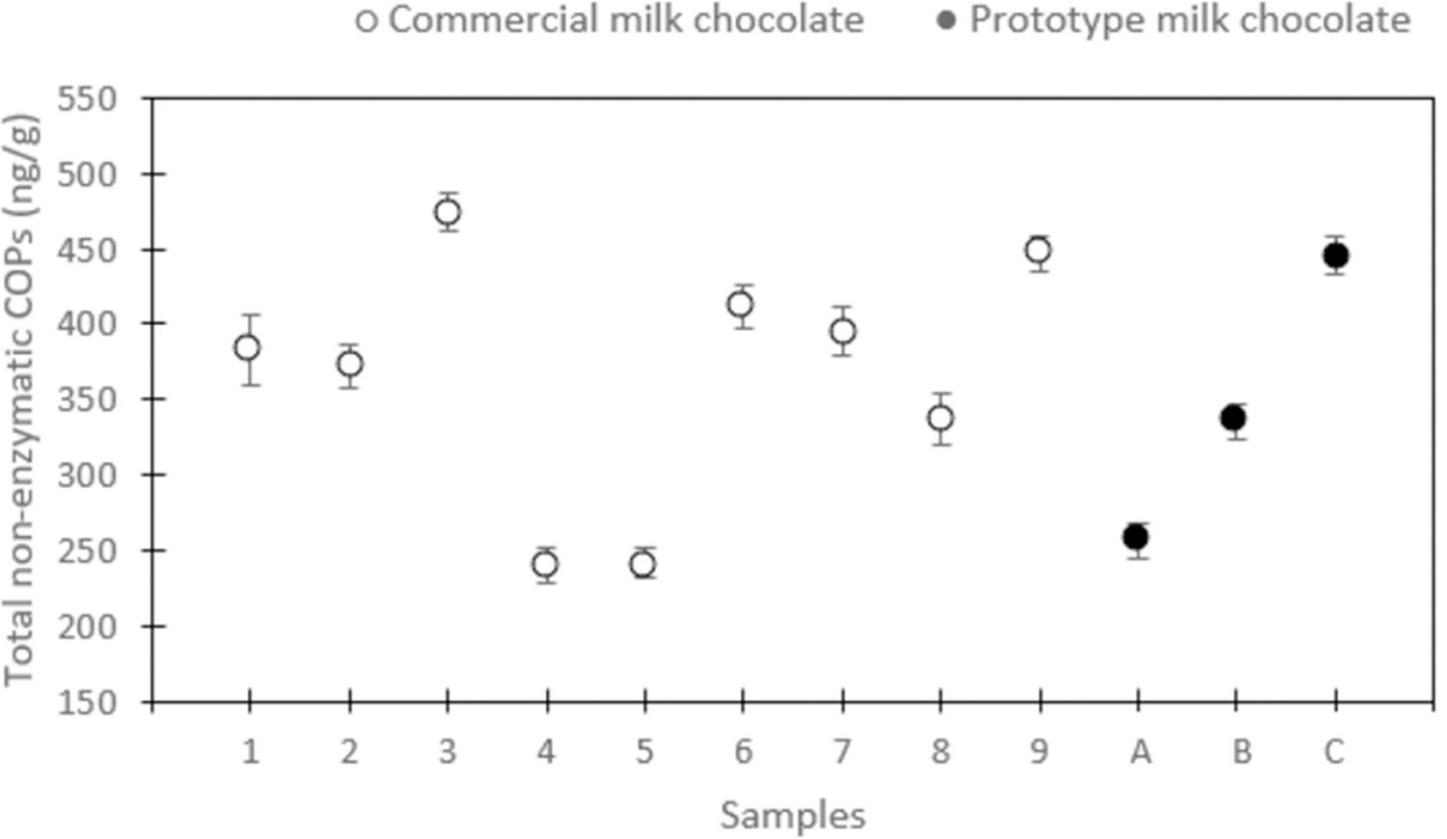
Distribution of non-enzymatic cholesterol oxidation products (COPs) in commercial and prototype milk chocolate tablets made with whole milk powder (WMP) of 20, 120, and 180 days of shelf-life, respectively). Commercial milk chocolate tablets: samples 1–9. Prototype milk chocolate tablets: sample A (20 days of shelf-life), sample B (120 days of shelf-life), sample C (180 days of shelf-life). Values are expressed as means ± SD (n = 3).

COPs has been reported in different cholesterol-containing foods but, in particular, powdered milk and other foodstuff at different shelf-lives [4, 21, 39, 45]. However, besides a few reports of biscuits and sweet snacks, data of finished products containing milk powdered ingredients are still lacking [15]. Quantitative data of POPs in chocolate bars and other composite products have however been reported [7, 11]. In the present work, for the first time, both enzymatic and non-enzymatic COPs were measured in commercial milk chocolate tablets. Total non-enzymatic COPs spanned from 240.79 ± 11.74 to 475.12 ± 12.58 ng/g, 27OHC from 27.70 ± 0.74 to 72.78 ± 1.74 ng/g, while the non-enzymatic COPs/cholesterol ratio ranged from 1.26 ± 0.02 to 2.18 ± 0.07 ng/μg (Fig 1 and Table 3).

**Table 3:** Ingredients, fat composition as indicated by the manufacturer, cholesterol (μg/g), and COPs (ng/g) content of the commercial samples of milk chocolate included in the study.

Chocolate samples	1.	2.	3.	4.	5.	6.	7.	8.	9.
**Ingredients**	sugar, cocoa butter, skimmed milk powder, cocoa mass, whey powder, *milk fat*, hazelnut paste, soy lecithin, flavoring	sugar, *whole milk powder (20%)*, cocoa butter, cocoa mass, hazelnut paste, soy lecithin	sugar, *whole milk powder*, cocoa butter, cocoa mass, soy lecithin	sugar, vegetable fat (coconut, palm kernel), cocoa butter, cocoa mass, *whole milk powder*, lactose, skimmed milk powder, *anhydrous butter*, soy lecithin, barley malt extract, flavorings	sugar, cocoa butter, *whole milk powder*, cocoa paste, skimmed milk powder, lactose, barley malt extract, soy lecithin, flavorings	sugar, *milk powder 26*,*4%*, cocoa butter, whey powder, sunflower lecithin, natural vanilla flavor	sugar, *whole milk powder*, cocoa butter, cocoa mass, whey powder, lactose, soy lecithin, vanilla extract	sugar, cocoa butter, *milk powder*, cocoa mass, powdered milk whey, sunflower lecithin emulsifier, vanilla flavor	cane sugar (39%), cocoa butter, *whole milk powder (23%)*, cocoa paste, vanilla pods
**Total fats (g/100)**	29.5	32	34.4	47	31	32	31	30	39
**Saturated fats (g/100)**	17.5	20	21	36	19	19	19	18	24
**Degree of unsaturation (%)**	41	38	39	23	39	41	41	40	38
**Cholesterol**	212.59 ± 3.24	237.11 ± 1.96	254.76 ± 2.71	117 ± 2.35	191.75 ± 6.18	321.26 ± 3.16	243.83 ± 8.45	163.99 ± 6.02	204.81 ± 1.15
**7β-hydroxycholesterol (7βOHC)**	169.45 ± 10.01	124.06 ± 3.23	253.33 ± 4.80	93.15 ± 2.24	107.55 ± 4.24	183.39 ± 4.67	228.46 ± 10.21	158.02 ± 8.54	251.43 ± 6.68
**7-ketocholesterol (7KC)**	146.20 ± 7.46	133.51 ± 6.55	141.27 ± 4.88	65.60 ± 2.55	50.19 ± 2.62	117.18 ± 3.41	94.92 ± 3.55	86.89 ± 3.78	90.68 ± 2.39
**5,6α-epoxycholestanol (α-epoxy)**	16.73 ± 0.69	17.75 ± 0.67	17.62 ± 0.29	12.31 ± 0.58	15.65 ± 0.37	18.25 ± 0.58	16.69 ± 0.71	17.89 ± 0.66	18.65 ± 0.13
**5,6β- epoxycholestanol (β-epoxy)**	14.48 ± 0.95	25.74 ± 2.08	19.85 ± 1.36	14.60 ± 0.98	23.94 ± 1.26	20.00 ± 1.6	24.29 ± 0.63	19.26 ± 0.68	18.77 ± 1.42
**cholestan-3β,5α,6β-triol (triol)**	4.41 ± 1.06	6.55 ± 0.15	3.16 ± 0.21	3.92 ± 0.39	6.76 ± 0.29	5.34 ± 0.33	5.17 ±0.49	4.31 ± 0.13	6.97 ± 0.49
**25-hydroxycholesterol (25OHC)**	31.85 ± 2.94	63.74 ± 1.56	39.90 ± 1.03	51.21 ± 5.00	37.35 ± 1.27	67.56 ± 3.39	26.30 ± 0.71	51.02 ± 3.08	60.09 ± 1.10
**27-hydroxycholesterol (27OHC)**	68.88 ± 2.20	66.77 ± 2.59	66.43 ± 3.86	27.70 ± 0.74	64.66 ± 2.01	72.78 ± 1.74	52.21 ± 1.17	62.77 ± 1.63	53.52 ± 1.41
**Total non-enzymatic COPs** ^1^	383.12 ± 23.12	371.36 ± 14.25	475.12 ± 12.58	240.79 ± 11.74	241.44 ± 10.05	411.73 ± 13.97	395.83 ± 16.30	337.40 ± 16.87	446.59 ± 12.23
**Non-enzymatic COPs** ^**1**^ **/cholesterol ratio (ng/μg)**	1.80 ± 0.12	1.57 ± 0.07	1.87 ± 0.04	2.06 ± 0.08	1.26 ± 0.02	1.28 ± 0.04	1.62 ± 0.02	2.06 ± 0.04	2.18 ± 0.07

Values are expressed as means ± SD (n = 3). Major cholesterol sources are in *italics*.

^1^ COPs: cholesterol oxidation products.

Similar to what observed in the case of WMPs and in the prototype chocolates, the main non-enzymatic COPs where 7βOHC and 7KC (69–83% of total COPs) (Table 3). The distribution of such oxysterols fell within the range of the three prototypical milk chocolates produced with milk powder ingredients at increasing shelf-lives (Table 2 and Fig 1). Without knowing the age and processing procedures of the ingredients used in the commercial chocolates, it is difficult to investigate the source of such difference. Indeed, COPs could arise from the production processes employed in the tablets manufacturing, and/or the quality of the raw materials used, considering that all the samples were produced from different manufacturers. A previous study performed on POPs presence in other composite products (i.e. spreadable creams), suggests however that the presence of these oxidative compounds could be attributed more to the oils used as raw materials, rather than the technological processes to produce them [44]. Similarly, it was shown how COPs content in biscuits reflected the quality and processing of egg products used as ingredients in the formulations [17]. In the case of milk chocolates, COPs amount would be a marker of the freshness and the quality of the milk powders used for their production: during normal dairy processing fat globules are vulnerable to disruption, increasing the susceptibility of cholesterol to oxidation [46]. COP quantities could also reflect the total fatty acid unsaturation pattern of the lipid fraction, as the presence of unsaturated fatty acids has been suggested to favor sterol oxidation [47]. While the commercial milk chocolate tablets analyzed in the current study presented a similar fatty acid unsaturation pattern, it is interesting to note, albeit without statistical significance, that the sample with the lowest fatty acid unsaturation pattern also presented the lowest amounts of total non-enzymatic COPs (p > 0.05) (Table 3). It is important to note that to fully validate the above-mentioned hypotheses, a detailed fatty acid unsaturation pattern as well as the analysis of possible antioxidant properties of food ingredients have to be planned in the continuation of the study. Literature data on POPs, however, strengthen this single observation, showing how differences in POPs could be explained based on their fatty acid composition, with more saturated vegetable oils containing lower POPs, being less prone to oxidation [48]. Indeed, the oxidative instability of cholesterol could be further favored by the presence of larger amounts of polyunsaturated fatty acids (PUFA) in the cell membrane: in real food systems, it has been shown that a higher unsaturation degree of the lipid matrix tends to promote cholesterol oxidation [10, 49]. Lastly, although this was not assessed by the present study, it must be noted that the presence and type of antioxidants could also affect cholesterol oxidation [50].

## Conclusion

COPs originating from animal-based sources, 7βOHC and 7KC in particular, have been confirmed as reliable biomarkers of food manufacturing, storage, and of the oxidative process more in general, and proposed as promising nutritional parameters and quality tools of powdered ingredients (milks and eggs, in particular). Although there is still a need of a systematic estimation of daily intake of COPs that would also distinguish between free and esterified oxysterols, a reduced consumption as a measure to dampen their formation and accumulation in processed foodstuff has been suggested, in light of the hypothesized undesirable health effects of such compounds at relatively high concentrations. For the first time, this work reports the presence of COPs in products of plant-based origin other than vegetable oils (i.e. cocoa butter, cocoa paste, and soy lecithin), contributing to informing on additional sources that could contribute, although in much lower amounts, to their dietary intake. In addition, quantitative data on COPs in composite products containing milk ingredients are still scarce. Here, it was shown how the total non-enzymatic COPs in commercial milk chocolates ranged from 240.79 ± 11.74 to 475.12 ± 12.58 ng/g, likely resulting from the technological processing and/or storage of the ingredients, the fatty acid unsaturation pattern, and/or the amount of antioxidants present in dose.

The results obtained from the prototypical milk chocolate tablets, however, point to the age of the milk powders as the main source of COPs in such products, compared to the quantity deriving from plant-based ingredients or from the technological processes of the finished products. While further studies confirming our findings and also quantifying the effect of different time/temperature processing conditions are needed, the results of this study represent a first application of non-enzymatic COPs as biomarkers of cholesterol autoxidation. Such could aid to further quantify and characterize the nutritional quality and freshness of milk powders not only as raw materials but also when employed in composite products, to potentially be used to substantiate the claims related to milk quality that these products often bear.
